# Hernias of the Utterine Appendages

**Published:** 1912-10

**Authors:** Aime Paul Heineck

**Affiliations:** Surgeon to the Cook County Hospital, Chicago


### HERNIAS OF THE UTERINE APPENDAGES.

By Aime Paul Heineck, M. D.,

*Surgeon to the Cook County Hospital, Chicago.*

In the female, the frequency of hernias and especially of hernias of the internal genitalia has been, and is still underestimated. Owing to the lack of study heretofore given to this clinical entity, hernias of the uterine adnexae are often overlooked, not uncommonly misdiagnosed, and therefore subjected to injudicious treatment: harmful alike to the individual and to the hernial contents, prejudiciaal alike to the patient's general well-being and to her reproductive powers. Impressed by the clinical importance of the condition and surprised at the insufficient consideration given to the subject in even the most modern gynecological and surgical text books, I have collected the following data which may prove of service to some of my professional colleagues as well as to future investigators of the subject. Knowledge of the occurrence and familiarity with the symptomatology of a clinical condition lead to its more frequent and more timely recognition.

Soon after beginning the consideration of the subject, we became convinced that deductions and conclusions to be valuable, should be based solely upon the study of cases accuracy of diagnosis is self-evident. Therefore in the preparation of this paper we have only considered cases in which the hernial contents were demonstrated at the dissecting, autopsy or operating table.

We have conformed to the nomenclature in actual use; however, to better insure precision of classification and a more intelligent discussion of the subject, we define, at times, perhaps needlessly, the terms employed. The word hernia signifies the permanent or temporary protrusion of one or more viscera from their normal situation through a normal or abnormal opening in the walls of the cavity within which they are contained. It implies the existence of a hernial ring, of a hernial sac, of hernial sac contents and of sac coverings. In the hernias discussed in this article, the protruding organ was always either an ovary, an oviduct, or Fallopain tube and an ovary. In some cases, as associated hernial contents we find omentum, a segment of the alimentary canal, a part of the urinary bladder, a rudimentary uterine horn or the entire uterus, be the latter organ rudimentary, infantile, or of normal development. The tube or ovary or both in part or in their entirety may be herniated. All the hernias herein considered are external hernias; that is, their outermost overlying saccular covering is skin, and each, after reaching a certain stage of development gives rise to a more or less visible, more or less palpable external swelling in the inguinal, femoral, ventral, obturator or ischiatic regions, depending upon the anatomical location of the hernia. Internal hernias, that is, hernias in which one or more loops of intestine find their way into pouches or recesses in the posterior peritoneal wall, and diaphragmatic hernias, be the latter true or false, congenital or acquired, constitue other chapters of surgery.

The escape of the uterine appendages from their normal situation may take place through any of the weak spots or openings of the lower abdominal or obdomino-pelvic cavities. A hernia originating either in the internal or in the external inguinal fossae and escaping above Poupart's ligament is an inguinal hernia; if escaping beneath the same ligament, it emerges through the crural canal and the saphenous opening, it is a femoral hernia; if through the obturator canal, an obturator hernia; if along the course of the gluteal or sciatic nerves and vessels, emerging almose always above, very infrequently below the pyriformis muscle, very rarely through the lesser sacro-sciatic foramen, a gluteal hernia; if through an operative scar in the abdominal wall, a post-operative hernia.

Though sanctioned by long usage, the classifying of hernias into congenital and acquired is, at times, misleading. It is misleading because it is practically impossible to determine the congenital or acquired nature of many hernias. Furthermore, the term congenital hernia, as now used, does not imply in the female that the hernia was present at birth, as it also applied to hernias whose post-natal development is due to predisposing conditions of congenital origin: developmental defects, persistence of transitory embryonal or fetal states which, owing to their non-disappearance with growth, permit the occurrence of outward visceral displacements. Some hernias are congenital in the truest sense of the word; they are complete at birth, hernial contents being then present. In most of the so-called congenital hernias, the sac only is existent at birth; in an acquired hernia, the sac is always of post-natal development, and in all but hernias par glissement is entirely derived from the parietal peritoneum. Congenital hernial sacs result from the want of closure of peritoneal processes, such as the processes vaginalis peritonei in the male, the canal of Nuck in the female, etc., normally present in the fetus. Congenital hernias may appear at any period of life.

Orifices for the transmission of vessels and ducts are normally present in the muscular and aponeurotic layers of the obdominal walls. An acquired hernia is formed by the gradual or sudden escape through these orifices, pathologically widened, of viscera normally contained within the abdominal cavity; the viscera in their passage through and beyond the abdominal wall create paths of escape for themselves by bulging and pushing forward the parietal peritoneum.

In many cases, the congenital or acquired nature of the hernia is either too vaguely stated or is left unmentioned. Femoral hernia seldom occurs before adult life.

Tubal, ovarian and tubo-ovarian hernias occur in the colored, and in the white race. Commonly, the condition is unilateral; infrequently, it is bilateral. When bilaterality, the hernias may or may not be developed to the same degree on both sides. The bilaterality may date from birth; may be acquired. In the latter case, the hernias may from the first have been bilateral or an interval of time shorter or greater length may have intervened between the appearance of the two hernias. In Broca's case, when patient was eight months old, the right inguinal hernia appeared, but the left inguinal hernia did not become manifest before the patient was four years of age. All the bilateral tubal, ovarian, or tubo-ovarian hernias recorded in the medical literature of the last twenty years are of the inguinal variety. This is in accord with a well-known fact that, in both sexes, double femoral hernias are less frequent than double inguinal hernias.

A tubal, an ovarian or a tube-ovarian hernia may co-exist with one or more hernas of other organs. Multiple hernias are not infrequent, the hernias present being either of the same or of different types, as two inguinal hernias or one inguinal and one femoral hernia on the same or on opposite sides of the body.

The hernias which we have under consideration may or may not be associated with non-development, malformation or absence of the other internal or of some external genitalia. In Martin's (Kessmann) case, a hernia of the left tube and ovary, the uterus was displaced upwards, forwards and to the left by a cyst of the right ovary having the volume of a man's head.

In almost all the cases in the operation performed was a herniotomy, and as herniotomy affords little opportunity for direct examination of the pelvic organs, the condition of the non-herniated genitalia was determined in only a few cases.

Hernis is a widespread disease. The relative incidence of hernias of the internal genitalia as to age corresponds to that of hernia in general. In our series, the youngest patient was four weeks old at the time of operation, Nicoll operated successfully for ovarian hernia, two infants each one and a half months old. The oldest patient operated on was seventy-eight years old. She had a left tubal oburater. Lickley reports a right tube-ovarian obturator hernia observed in a dissecting room subject who had died of general debility and hemiplegia at the age of eighty-seven years. In many cases, the age is not stated. The age given by most authors corresponds to the age of the patient at the time of operation and not to the age at which the hernia first appeared.

The study of the reported cases demonstrates:—

(a). The frequency of hernias during the first year of life.

(b). The rarity of the condition from the first to the fifteenth year of life.

(c). The noticeable progressive increase in the number of hernias observed from the fifteenth year on, the maximal frequency being seen during the fourth decade of life.

(d). After the fortieth year, there is a decline of the number of hernias; they become relatively rare as the extremes of life are approached.

During the child-bearing period, hernias of either or both uterine appendages have been observed in nulliparae, in primiparae and in multiparae.

### Etiology.

The predisposing and exicting causes of tubal, ovarian and tube-ovarian hernias are shown by the analysis of the collected cases to be the same as those of other hernias in the female. The persistence of the canal of Nuck is an etiological factor of the greatest significance in the causation of inguinal hernias. The canal of Nuck, the homologous of the precessus vaginalis peritonei in the male, is a peritoneal diverticulum accompanying and adhering intimately to the round ligament, descending in some cases as far as the insertion of that ligament in the labium majus.

This peritoneal process, whose dates of origin and disappearance are not accurately known, is usually found completely obliterated at birth, it may close after birth; it may even persist throughout life. When the canal of Nuck is only partially or completely unobliterated, it forms a potential hernial sac and is conceded to be the most important congenital predisposing cause to inguinal hernia formation. It is a matter of common knowledge that preformed sacs are not of infrequent occurrence in the inguinal region. They have been found in other hernial regions. A sudden or forcible increase in intra-abdominal pressure, such as can be determined by muscular effort, by a misstep in an attempt to save one's self from falling, can lead to hernia formation by causing the interruption in a preformed sac of a tube, an ovary or a tube and ovary. As during infancy, the internal genitalia can neither be displaced by their physiological activity which is nil, nor by the development of pelvic tumors (rare during early childhood), nor by muscular effort, it follows that hernias of the uterine appendages, at that period of life, are due to such congenital anatomical defects as facilitate tubal, ovarian or tubeovarian displacement.

Though, as an etiological factor in the production of hernias, the existence of hereditary predisposition is denied by some authors, to us, the influence of hereditary appears positive. Hernia being a malformation often due to developmental arrest, such as non or incomplete obliteration of the processus vaginalis peritonei, non-obliteration of umbilical ring, etc., it is subject to hereditary transmission. It is reasonable to assume that like structural characteristics beget alike predisposition and a like resistance to hernia development. Among other etiological factors should be mentioned:-

I. *All conditions associated with increased mobility of the uterine appendages.*

(a). Lengthening of the broad ligaments consecutive to repeated pregnancies. Owing to its loose attachment to the broad ligament, the position of the ovary is easily affected by the physiological or pathological movements and displacements of the pelvic organs.

(b). Pathological relaxation of the ligaments due to puerperal sub-involution. As a cause of the various uterine displacements, pregnancy is an important factor.

(c). Abnormal length of the broad, ovarian and infundibule-pelvic ligaments.

(d). Relaxation of the other tubal, ovarian and uterine ligaments.

II. *All conditions that tend to increase the intra-abdominal pressure*:-

(a). By overcoming the resistance offered by one or another of the weak points of the abdominal wall. Sudden increase of the intra-abdominal pressure may lead to the irruption of a tube, ovary in the sac of an old enterocele.

(b). Occupations necessitating repeated muscular efforts associated with increased intra-abdominal tension, as the lifting or pushing of heavy weights, etc.

(c). Physiological or pathological states which distend the abdominal cavity, which stretch the abdominal parietes and widen the orifices normally present in the muscular and apeneurotic layers of the abdominal wall. Enteroptosis, obesity, abdominal tumors, ascites, pregnancy, etc., can be regarded as predisposing and exciting causes to hernia production.

III. *All conditions which weaken the abdominal wall. A hernia can occur whenever the parietal peritoneum is not sufficiently supported by the transversalis fascia and the other structures of the abdominal wall.*

(a). Acute or chronic diseases debilitating the organism, especially such as cause great emaciation.

(b). Obesity weakens the abdominal pressure.

(c). Traumatism.

(d). Feeble development, epiploceles and entero- epiploceles.

(e). Feeble development or atrophy of the aponeuresis of the transversalis muscle and of the conjoined tendon.

(f). A shortening of the round ligament of the hernial side is not rare.

### HERNIAL SACS.

Congenital hernias have sacs of pre-natal formation. That the canal of Nuck remains patent in many cases long after birth, even into adult life, has been proven by a number of investigators. Acquired hernial sacs are formed of parietal peritoneum forced by intra-abdominal pressure through some congenital or acquired defect in the abdominal walls.

In the female the following anatomical characteristics are strongly suggestive of the congenital sacs. Great vascularity, absence of sub-serous fat, folds, valvular or diaphragmatic constructions, cyst-formations, scar-like induration of the wall, etc. In congenital inguinal hernias, it is also noted that the round ligament is intimately adherent to the sac.

As we see, the sac results from the bulging outward of the parietal peritoneum without solution of continuity. It consists of a neck, body and fundus. Of these three, the neck is the most important. It is through it that the general peritoneal cavity communicates with the hernial sac cavity. It may be the site of constriction. The fibrous envelope of the sac is due to the condensation of the fibrous layers which the hernia in developing pushes forward.

The internal surface of the sac, whether the latter be congenital or acquired, has all the peculiarities of serous membranes.

In the female, encysted inguinal hernias result from partial closure of the canal of Nuck, the peripheral portion of which is transformed into a cyst in which a developing hernia prolapses or from the sinking of a hernia in pre-existing hernial hydrocele.

There is really no sacless hernia; but the herniated viscus may be an organ only partially covered with peritoneum.

In sliding hernias, the anterior and lateral portions of the sac are, as in ordinary hernias, derived from the parietal peritoneum, while the remaining portion is formed by the anterior surface of the herniated caecum, sigmoid, bladder or Fallopian tube.

Hernial sacs may be dome-shaped, cylindrical, digitiform, sacculated or irregular; may show constrictions with intervening dilated portions. Most femeral sacs are small and not infrequently are embedded in a mass of fat. The sac in recent hernias may be very thin. In children and in young infants, sac is extremely thin.

### HERNIAL FLUID.

The reporters not infrequently state that fluid of some nature or other was present in the hernial sac. The fluid may be serous, sanious, muco-purulent, purulent or fetid in character.

### HERNIAL SAC-CONTENTS.

### TUBAL HEBNIAS.

The tube, either in part or in its entirety, may be the sole content of the hernial sac. As associated hernial contents, a portion of the urinary bladder, normal intestine, gangrenous intestine and omentum were noted.

The herniated tube may be normal and free; may be adherent to sac; may be strangulated; may show inflammatory lesions; may be cystic; may be the seat of a pyosalpinx.

### OVARIAN HERNIAS.

The ovary may be the only content of the hernial sac; or may be associated with a cystic parovarium, with omentum, with intestine or with a rudimentary uterus. The herniated ovary is reported as being in some cases normal, in others enlarged to twice its normal size, infiltrated with blood, the seat of a large hematoma, adherent to sac, cystic, to have presented areas of suppuration, to have shown gangrenous changes.

### TUBO-OVARIAN HERNIAS.

Hernias of the tube and ovary constitute the largest number of hernias of the uterine appendages. As associated hernial contents may be mentioned:- Urinary bladder, Meckel's diverticulum, the appendix veriformis, omentum, intestine and the uterus.

We know of onl two cases in which gestation occurred in an inguinal hernial sac; both were tubal pregnancies.

The herniated organs may be normal, may show slight or marked pathological changes. The displacement of a tube or of an ovary into a hernial sac is unfavorable to its anatomical and functional integrity. In a hernial sac, these organs are exposed to repeated slight traumatisms and to circulatory disturbances. The herniated ovary frequently undergoes cystic changes; may show atrophy; may show enlargement; may be undersized.

The herniated tube may be the seat of suppurative salpingitis, of abscess, of tuberculosis; may be adherent to sac by an inflammatory band.

We must not forget to state that a tube and ovary present in a hernial sac do not always have the same reciprocal relation that they have in the abdominal cavity, and that the pathological changes which they show may antedate their displacement into a hernial sac.

### Reducible Hernias.

In reducible hernias, and, at first, practically all hernias are reducible, the hernial contents either return spontaneously into the abdominal cavity when the patient assumes the recumbent posture or they can be manipulated back, with more or less difficulty, but without a cutting-operation, into the cavity from which they escaped. Even in reducible hernias, the sac early contracts adhesions to neighboring tissues and becomes irreducible. The terms reducibility, irreducibility, torsion and strangulation have reference only to the hernial contents and not to the sac.

Reposition, spontaneous or manual, may be temporary, may be permanent. Many reducible hernias reappear as soon as the standing posture is assumed; others, to reprotude require more or less muscular effort on the part of the patient.

### Irreducible Hernias.

When the contents of a hernial sac cannot in their entirety be manipulated back into the abdominal cavity, the hernia is said to be irreducible, provided that there is not any or but a very slight interference with the blood supply of the herniated organ or organs, and that there is no disturbance of function. If irreducibility, and both functional and circulatory disturbances are present, partial or complete, predispose to complications of a serious nature:- Inflammation, incarceration, strangulation and torsion.

The irreducibility of hernias is dependent upon one or more of the following factors:-

(a). Difficulties incident to manipulating a small movable body such as the ovary through a small opening.

(b). Relative narrowness of the hernial canal:- Femoral, inguinal, etc.

(c). Sudden increase in size of hernia resulting from some unusual muscular effort.

(d). Changes in the hernial contents:- Increase in bulk from deposit of fat, from cyst formation in mesentery, in omentum, from inflammatory or neoplastic changes.

(e). Adhesions of inflammatory origin:-

I. *Between sac and contents;*

II. *Between the different contents.*

(f). Large volume of the hernia.

(g). Sliding hernias (Hernias par glissement).

The irreducibility of a hernia of the uterine appendages i. due in some cases to the presence as associated hernial contents of the urinary bladder, of the caecum, or of the sigmoid. In other cases, the irreducibility is due to the fact that the layers of the broad ligament, as they leave the Fallopain tube, enter into the formation of the hernial sac.

STRANGULATED HERNIAS.

All strangulated hernias are irreducible. In addition to irreducibility, they present a constriction of the hernial contents of such a degree as to seriously interfere with the circulation ol the blood in the hernial organ or organs. "If the pedicle of a tumor is tied off or if a finger is surrounded tightly by a string, the parts distal to the ligature do not become inflated; stasis and gangrene result" (Alberts). The same primary changes occur in the contents of a strangulated hernia; the inflammatory changes are secondary to the circulatory disturbances. There is interference first with the venous circulation; then with the arterial. As a result of this interference with the circulation, we have a serous exudate the amount of which depends upon the degree and duration of the strangulation and also upon the extent of the secreting surface. The sequence of events is as follows:- Congestion, stasis, serous exudation, then inflammatory phenomena and gangrene. Strangulation of a herniated tube or ovary is not as dangerous a complication as strangulation of a herniated loop of intestine.

Strangulated inguinal hernias may be congenital or acquired. Strangulaton can occur at any age, irrespective of type of hernia or of hernial contents.

As hernial contents can be mentioned the following organs:- Fallopain tube, ovary, tube and ovary, tube and a portion of the urinary bladder, tube and loop of small intestine, ovary and intestine, tube, ovary, intestine and omentum, uterus and adnexae of one side, loop of intestine, uterus and left adnexa.

The symptoms given by the strangulated inguinal hernias are those of inflamed hernias. Symptoms of intestinal obstruction may also be present.

### Torsion of the Pedicle.

This complication, peculiar to ovarian and to tube-ovarian hernias, is not of unusual occurrence. As far as we have been able to determine, torsion of the pedicle has been observed only in irreducible congenital hernias of the inguinal type.

The two youngest patients were four and eleven weeks old respectively; the oldest, a poorly nourished child, was fourteen months old. All the other patients were less than one year old. The right and left side are involved with about the same frequency. The occurence of this accident is favored by the mobility of the ovary and the slenderness, at this period of life, of the pedicle of the herniated organ or organs.

The pedicle, usually composed of the Fallopian tube, broad ligament and contained vessels, may have made a half-turn upon itself; may be twisted twice, thrice or several times upon its axis. The pedicle may be twisted in any part of its course.

If unrelieved, torsion of the pedicle determines in the hernial contents anatominal changes similar to those caused by strangulation. The impeded return of blood in the veins leads to the congestion and swelling of the organ or organs below the twist. There are noticed in the hernial contents, the following circulatory disturbances:- Congestion, statis, thrombosis, vascular rupture (ovary seat of large hematoma) and intestinal hemorrhages. The interstitial hemorrhages and the serous transudates lead to tissue dissociation.

In torson of the pedicle, the amount, odor and color of the hernial fluid depends upon the tightness and duration of the twist and upon the extent of the gargerenous changes. The fluid present in the hernial sac may be serous, may be blood-stained, dark colored, reddish-brown.

Torsion of the pedicle gives rise to symptoms somewhat anologous to those of strangulated intestinal or omental hernias. In fact, the condition has frequently been diagnosed a strangulated intestinal hernia.

### Post-Operative Ventral Hernias or Hernias in Abdominal Scars.

The protrusion of parietal peritoneum with stretching of the cicatrix over it, may occur after any operative or other penetrating wound of the abdominal wall, except those of very small dimensions.

Though these hernias may occur in any part of the abdominal wall, they are located almost always either in the median line or in the region of the appendix. Owing to the employment of improved operative technique, and to the more rigid observance of the requirements of surgical asepsis, post-operative hernias are decreasing in frequency.

There are two types:- In one, there is a uniform distention of the cicatricial tissue producing a condition somewhat analogous to separation of the recti muscles; in the other, the hernia is due to the giving way of weaker portions of the scar.

The main predisposing etiological factors of post-operative hernas are:

1. Long incisions.
2. Faulty closure of abdominal wounds.
3. Operations for suppurative processes which of themselves require, for healing, that the abdominal wound be maintained open for a considerable period of time.
4. Drainage.
5. Disturbed wound healing-Imperfect asepsis-Suppuration.
6. Failure to wear for some months after recovery from operation, a well-fitting abdominal binder.
7. Too early pressure on the scar.

8. Pregnancy.

The only ventral hernia of the uterine appendages which we could find is a tube-ovarian hernia in an abdominal scar resulting from the opening and prolonged drainage, ten years previously, of an appendiceal abscess. The scar was about six inches in length. Cullen, to cure this case, resected the entire cicatrix, loosened the adhesions of the omentum to the ovary; then removed the herniated ovary and hydro-salpinx and followed this by closure of the abdominal wound without drainage. A satisfactory recovery was obtained. The sac was incomplete for a considerable distance; the ovary lay directly beneath the skin.

### Gluteal, Sciatic or Ischiadic Hernias.

This is a very uncommon condition; in the medical literature of the last one hundred and fifty years, only twenty-three cases are recorded (E. Koeppl). This class of hernias escape from the abdominal cavity by way of either the greater or lesser sacro-sciatic foramen. There are three varieties:- The supra-pyriformis, the infra-pyriformis and the spine-tuberosa. These hernias may be congenital or acquired, may occur on either side of the body and are subject to all the complications of hernia in general. Thirteen of the cases on record were observed in women. Schilbach's case is the only one on record making its exit through the lesser-sacro-sciatic foramen. The diagnosis was first made at the autopsy table. During life, there had been genital hemorrhage and symptoms of ileus. At the post-mortem examination, the ovary was found in the hernial sac, the tube and broad ligament being caught in the hernial ring.

We found only two cases of ischiadic hernia of the uterine appendages. Both were acquired hernias of the right tube and ovary, occurring in multiparous patients. Both were subjected to operation and recorded.

Woelfler's case in interesting in that it was successfully subjected to an operation for radical cure. The hernia, an infra-pyriformis one, emerged like all others of its type along the lower border of the pyriformis muscle in close relation to the internal pudic, inferior gluteal and sciatic nerves and vessels. For the previous two years, the patient had had attacks of pain radiating along the course of the sciatic nerve, and abdominal suffering associated with nausea and, at times, vomiting. In the right gluteal region, there could be palpated below the muscles, a globular, fluctuating, fist-sized-, non-reducible swelling from which, at one time, there was removed by aspiration fifty cubic centimeters, and, at another time, five hundred cubic centimeters of dark reddish fluid containing much albumen, red blood corpuscles and leucin-tyrosin crystals.

The following operation was performed:- An eight centimeter long incision, parallel to the course of the fibres of the gluteus maximus, was made over the summit of the hernial swelling. The muscle fibres were separated; the hernial sac exposed, isolated and opened. It contained the ovary and the end of the tube. The sac contents were ablated; the resulting stump reduced in the abdominal cavity. The sac was then ligated and cut off; after which, the operator closed the hernial orifice by approximating the pyriformais muscle to the lesser sacro-sciatic ligament.

### Obturator Hernias.

In obturator hernias, the herniated viscus or viscera, always, escape from the abdominal cavity by way of the obturator or sub-pubic canal.

These hernias, though less infrequent than ischiadic hernias, are nevertheless uncommon; not more than two hundred cases are recorded in the medical literature.

They are usually small; may be unilateral or bilateral (are more frequent on the right side); may co-exist with hernias of a different type. They may be reducible, irreducible or strangulated.

Pisque and Poirier recognize three main anatomical varieties of obturator hernia. In the first variety, the most common, the hernia follows the entire course of the obturator canal, appearing as a swelling in front of the external orifice of this canal.

In the second variety the hernia escapes from the abdominal cavity through the pelvic orifice of the sub-pubic canal, but following the course of the inferior division of the obturator nerve, does not traverse the canal's entire length, and makes its exit by passing between the superior and middle bundles of the obturator externus muscle. In the third variety, the hernial protrusion also enters the pelvic orifice of the obturator canal, but becomes lodged between the obturator membrane and the obturator externus muscle.

Objective symptoms are frequently absent. When a swelling is visible and palpable, it is usually of small volume and is located in the most internal portion of Scarpa's triangle, somewhat resembling a femoral protrusion. It is, however, non-pediculated and does not extend in the direction of the crural canal. In suspected cases, one should always determine whether there is increased pain when the obturator externus is put under tension. Abduction and rotation inward of the thigh.

Vaginal examination is important. The internal orifice of the sub-pubic canal is accessible to the vaginal hand.

Two routes are advised for the treatment of obturator hernias:- The abdominal route and the obturator route. In the obturator route, the following steps are employed.

1. An incision eight centimeters long is made about three and a half centimeters internal and parallel to the femoral artery.

2. Separate with a grooved sound the internal border of the pectineus muscle from the outer border of the abductor brevis and adductor longus muscles.

3. If necessary, divide a few of the fibres of the pectineus muscle close to their insertion on the pubic bone, so as to facilitate digital exploration of the obturator region.

4. Expose, isolate and open sac; determine its relation to the obturator nerve and vessels, after which nick constricting point if hernia be strangulated and reduce hernial contents.

5. Closure of wound.

*(To be continued).*

